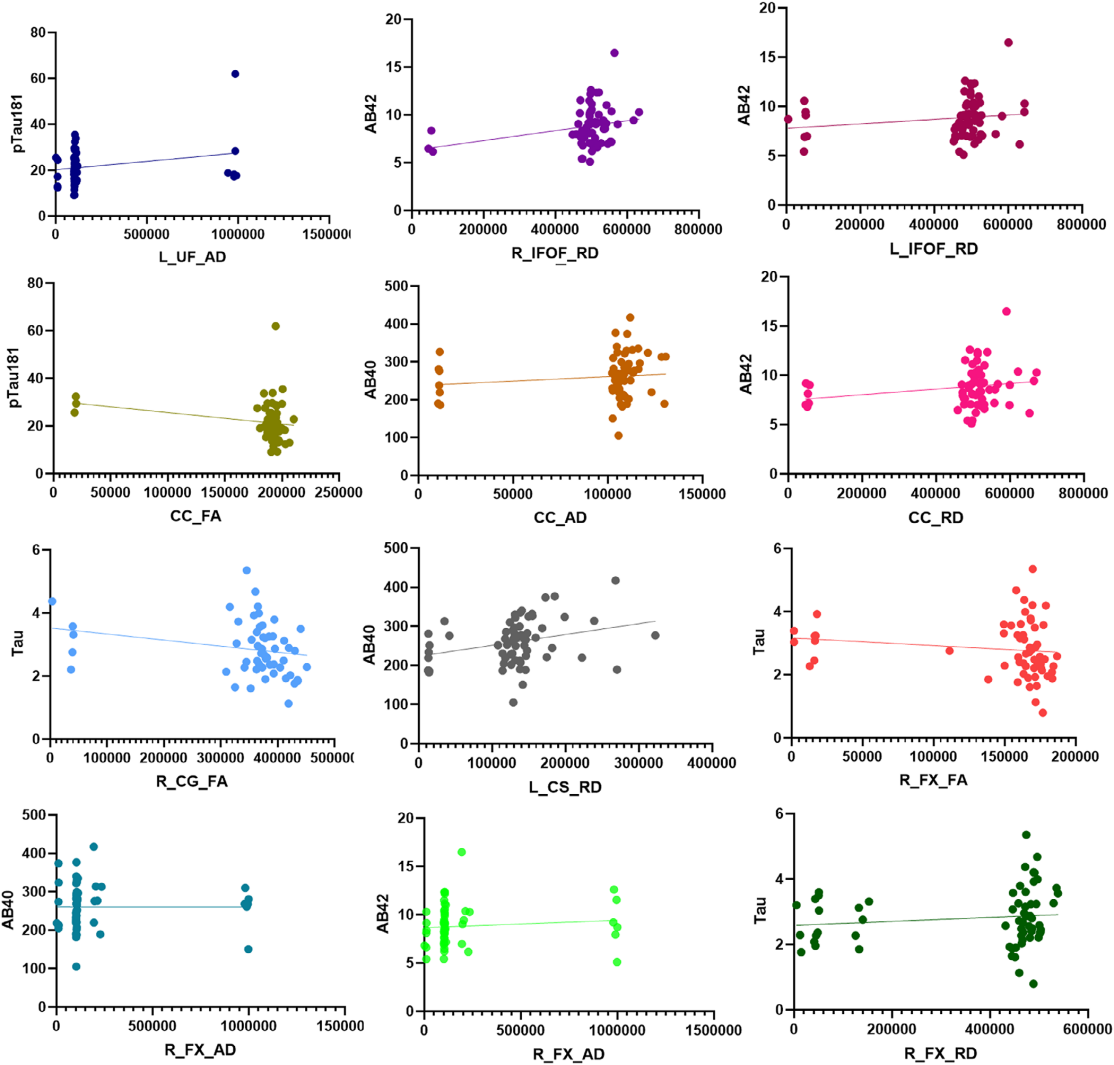# Plasma biomarkers are related to white matter integrity in alzheimer's disease continuum

**DOI:** 10.1002/alz70856_106760

**Published:** 2026-01-07

**Authors:** Isadora Cristina Ribeiro, Brunno Machado de Campos, Liara Rizzi, Luis E. Santos, Ananssa Silva, Thaís Lopes Pinheiro, Ítalo Karmann Aventurato, Brenda Costa Gonçalves, Marjorie Cristina Rocha da SIlva, Fernanda Guarino De Felice, Fernando Cendes, Marcio Luiz Figueredo Balthazar

**Affiliations:** ^1^ Universidade Estadual de Campinas (UNICAMP), Campinas, SP, Brazil; ^2^ Memory and Aging Center, UCSF Weill Institute for Neurosciences, University of California, San Francisco, San Francisco, CA, USA; ^3^ D'Or Institute for Research and Education, Rio de Janeiro, RJ, Brazil; ^4^ UNICAMP ‐ UNIVERSIDADE ESTADUAL DE CAMPINAS, CAMPINAS, SP, Brazil; ^5^ Unicamp, Campinas, Brazil; ^6^ School of Medical Sciences/University of Campinas (UNICAMP), Sao Paulo, SP, Brazil

## Abstract

**Background:**

Plasma biomarkers (PB) of Alzheimer's disease (AD) is a non‐invasive and economic alternative for early diagnosis. Due to the increasing incidence of dementia worldwide, understanding the relationship between PB and other AD biomarkers, such as neuroimaging is necessary. However, it is unclear whether PB concentrations reflect changes in brain anatomy observed on MRI. This study investigated the correlation between AD BP and white matter integrity.

**Method:**

63 older adults in AD continuum (42 Mild cognitive impairment, 8 Subjective cognitive decline, 13 Control) underwent magnetic resonance imaging examination and blood collection. Plasma samples were aliquoted and stored in cryogenic tubes in a gallon of nitrogen. Single Molecule Array was used to investigate the concentrations of BA40, BA42, Tau, and pTau181 in blood samples. Diffusion tensor imaging parameters (fractional anisotropy (FA) and axial (AxD) and radial diffusivity (RD)) were assessed with a preprocessing script that merges MRTrix, FSL, ANTs, and SPM12 tools. We investigate tracts related to AD (uncinate fasciculus (UF), inferior frontal‐occipital fasciculus (IFOF), corpus callosum (CC), corticospinal tract (Cs), cingulum (Cg) and fornix (Fx). Due to the nonparametric data, we used Spearman's correlation (*p* < 0.05; SPSS 26).

**Result:**

We found a positive correlation between plasma pTau181 concentrations and the AxD value in the left UF (r=0.273, *p* = 0.03), of AB42 with RD in the right and left IFOF (r=0.255, *p* = 0.04; r=0.263, *p* = 0.03) and in the CC (R=0.261, *p* = 0.03). In addition, BA42 with AxD in the right Fx (r=0.334, *p* = 0.00). AB40 correlated positively with AxD in the CC (r=0.259, *p* = 0.04) and in the right Fx (r=0.271, *p* = 0.03) with RD in the left Cs (r=0.334, *p* = 0.00). And Tau with RD in the right Fx (r=0.296, *p* = 0.01). In addition, negative correlations between FA values in the CC with ptau181 (r=‐0.292, *p* = 0.02), with tau in the Right Cg (r=‐0.318, *p* = 0.01) and right Fx (r=‐0.315, *p* = 0.01).

**Conclusion:**

We observed that higher concentrations of AD PB are associated with demyelination and axonal degeneration (such as AxD and RD), while lower concentrations of tau and ptau181 reflect low axonal degeneration and demyelination. PB reflects changes in white matter integrity.